# 415. Optimizing Blood Culture Utilization in Solid Organ Transplant Recipients: A Diagnostic Stewardship Approach

**DOI:** 10.1093/ofid/ofae631.129

**Published:** 2025-01-29

**Authors:** Julie M Steinbrink, Nitin Mehdiratta, Heather E Pena, Ian Welsby, Jessica Seidelman, Manuela Carugati

**Affiliations:** Duke University, Durham, NC; Duke University Hospital, Chapel Hill, North Carolina; Duke University Hospital, Chapel Hill, North Carolina; Duke University, Durham, NC; Duke University School of Medicine, Durham, North Carolina; Duke University, Durham, NC

## Abstract

**Background:**

Diagnostic stewardship of blood culture utilization is paramount to mitigate the risks associated with excessive culturing, including increased contamination rates and inappropriate antimicrobial administration. Although blood culture algorithms have been studied previously, there is insufficient data on their application specifically in solid organ transplant recipients. This study aims to retrospectively apply a blood culture algorithm (initially developed for a non-immunocompromised population) to assess its effectiveness in this immunosuppressed population.

**Figure 1.**
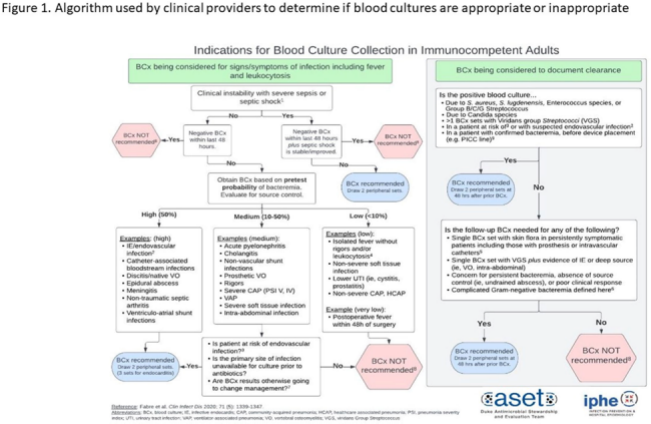
Algorithm used by clinical providers to determine if blood cultures are appropriate or inappropriate

**Methods:**

We conducted a retrospective review of adult solid organ transplant recipients with a blood culture event (BCE) occurring between February 2022 to January 2024 at a single academic medical center. A BCE was defined as the collection of at least one blood culture set for a specific clinical indication. During this time a blood culture algorithm (Figure 1) was implemented across 12 units in the hospital. Patient charts were manually reviewed to categorize BCEs as appropriate (the algorithm agreed with obtaining a blood culture), inappropriate (if the algorithm suggested to not draw a blood culture), or lacking documentation.

**Figure:**
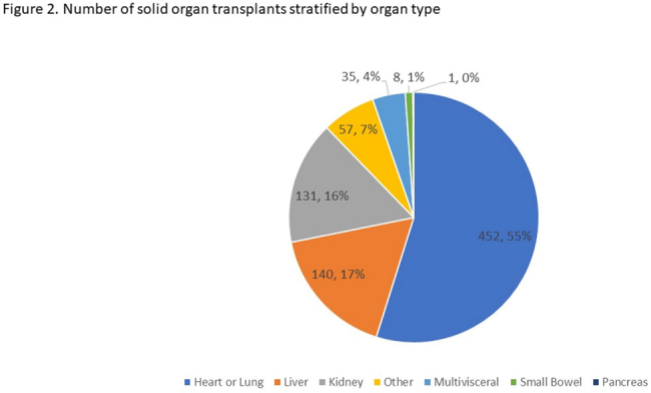

**Results:**

Among 824 BCEs recorded in adult solid organ transplant recipients (Figure 2), 669 (81.2%) were deemed appropriate, 118 (14.3%) were inappropriate, and 37 (5.5%) lacked sufficient documentation. Adjudication of 388 BCEs with available data was performed and revealed 350 (90.2%) negative cultures, 25 (6.4%) true positives, and 13 (3.4%) contaminants (Figure 3). Within the subset of inappropriate BCEs (n=118), 113 (95.8%) yielded negative cultures, while 5 (4.2%) were contaminants; no true positives were identified.

**Figure:**
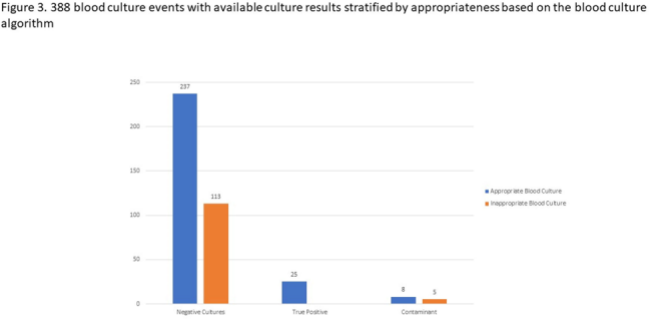

**Conclusion:**

The retrospective application of the blood culture algorithm did not reveal any missed true positive bacteremias in solid organ transplant recipients. Although this study is limited in scope, it provides initial evidence supporting the cautious application of the algorithm in this population. As a next step, we will adjudciate the remaining 436 BCE in the dataset. Further investigation is warranted to validate these findings and optimize diagnostic stewardship strategies for improved patient outcomes.

**Disclosures:**

**Jessica Seidelman, MD, MPH**, 3M: Expert Testimony

